# Virus and Viroid-Derived Small RNAs as Modulators of Host Gene Expression: Molecular Insights Into Pathogenesis

**DOI:** 10.3389/fmicb.2020.614231

**Published:** 2021-01-14

**Authors:** S. V. Ramesh, Sneha Yogindran, Prabu Gnanasekaran, Supriya Chakraborty, Stephan Winter, Hanu R. Pappu

**Affiliations:** ^1^ICAR-Central Plantation Crops Research Institute, Kasaragod, India; ^2^School of Life Sciences, Jawaharlal Nehru University, New Delhi, India; ^3^Department of Plant Pathology, Washington State University, Pullman, WA, United States; ^4^Leibniz-Institut DSMZ–Deutsche Sammlung von Mikroorganismen und Zellkulturen GmbH, Braunschweig, Germany

**Keywords:** gene regulation, off-targets, pathogenicity factor, plant–virus interactions, small RNAs, vsiRNA, vd-siRNA

## Abstract

Virus-derived siRNAs (vsiRNAs) generated by the host RNA silencing mechanism are effectors of plant’s defense response and act by targeting the viral RNA and DNA in post-transcriptional gene silencing (PTGS) and transcriptional gene silencing (TGS) pathways, respectively. Contrarily, viral suppressors of RNA silencing (VSRs) compromise the host RNA silencing pathways and also cause disease-associated symptoms. In this backdrop, reports describing the modulation of plant gene(s) expression by vsiRNAs via sequence complementarity between viral small RNAs (sRNAs) and host mRNAs have emerged. In some cases, silencing of host mRNAs by vsiRNAs has been implicated to cause characteristic symptoms of the viral diseases. Similarly, viroid infection results in generation of sRNAs, originating from viroid genomic RNAs, that potentially target host mRNAs causing typical disease-associated symptoms. Pathogen-derived sRNAs have been demonstrated to have the propensity to target wide range of genes including host defense-related genes, genes involved in flowering and reproductive pathways. Recent evidence indicates that vsiRNAs inhibit host RNA silencing to promote viral infection by acting as decoy sRNAs. Nevertheless, it remains unclear if the silencing of host transcripts by viral genome-derived sRNAs are inadvertent effects due to fortuitous pairing between vsiRNA and host mRNA or the result of genuine counter-defense strategy employed by viruses to enhance its survival inside the plant cell. In this review, we analyze the instances of such cross reaction between pathogen-derived vsiRNAs and host mRNAs and discuss the molecular insights regarding the process of pathogenesis.

## Introduction

RNA-triggered gene silencing is a highly conserved mechanism in eukaryotes. The key feature of RNA silencing is the production of 21–25 nucleotides (nt) long small RNAs (sRNAs) (Hamilton and Baulcombe, 1999; Brodersen and Voinnet, 2006) downregulating the expression of cognate mRNAs. The various classes of plant sRNAs attributed with regulatory functions are small interfering RNAs (siRNAs), microRNAs (miRNAs), natural antisense transcript-derived siRNAs (natsiRNAs), heterochromatic siRNAs (hcsiRNAs) or repeat-associated siRNAs (rasiRNAs), and long siRNAs (lsiRNAs) (Chapman and Carrington, 2007).

The trigger for RNA silencing is either double stranded RNA (dsRNA) or hairpin RNA (hpRNA) or fold-back structures formed by ssRNAs derived from the pathogens. These RNA structures are recognized by the plant host-derived ribonucleases, Dicer-like (DCL) enzymes, which cleave the dsRNA into siRNA duplexes (Bernstein et al., 2001). The Arabidopsis genome is known to encode four different DCLs producing siRNAs of varied sizes. DCL4 generates 21-nt-long siRNAs that confer antiviral resistance against RNA viruses, and also produces *trans*-acting siRNAs (ta-siRNAs), involved in endogenous gene regulation. Resistance against DNA viruses and regulation of transposons/or repetitive elements are conferred by 24-nt siRNAs produced by DCL3, whereas DCL2 produces 22-nt siRNAs and coordinates with DCL4 in conferring antiviral response. DCL1 is involved in the production of endogenous sRNAs, called miRNA duplex, in the nucleus (Hamilton et al., 2002; Tang et al., 2003). The resultant sRNAs are recruited by the Argonaute (AGO) proteins in the RNA-induced Silencing Complex (RISC). In general, AGOs of flowering plants regulate developmental and stress-related pathways, and maintain genome integrity. Plant AGOs are phylogenetically classified into three major clades, namely, *AGO* 4/6/8/9, *AGO* 1/5/10, and *AGO* 2/3/7. Among them, *AGO* 1/5/10 proteins bind to 21-nt miRNAs and siRNAs and are primarily involved in post transcriptional gene silencing (PTGS). *AGO* 2/3/7 interact with vsiRNAs, miRNAs, and most importantly tasiRNAs and are involved in plant defense and morphogenesis. *AGO* 4/6/8/9 proteins bind to 24-nt siRNAs and other endogenous siRNAs and recruit DNA methyltransferase *DRM2* to effect RNA-dependent DNA methylation (RdDM) (Zhang et al., 2015). The RISC, of which AGO is an integral component, slices the target mRNA in a sequence-specific manner. Moreover, siRNAs are involved in transcriptional gene silencing (TGS) by RNA-directed DNA methylation (RdDM) affecting the genomes of DNA viruses (Raja et al., 2010; Coursey et al., 2018) and in the histone modification of plant genome.

In addition to RNases/slicers like AGOs, host-derived RNA-dependent RNA polymerases (RdRPs/RDRs) also play major roles by producing secondary siRNAs (Mourrain et al., 2000; Vaistij et al., 2002). The RDRs could generate the dsRNA using ssRNA as a template either in a primer-dependent or primer-independent manner (Tang et al., 2003; Qi et al., 2009). The secondary siRNAs ensure that the silencing is amplified, suggesting the participation of RDRs in vigorous maintenance of RNA silencing even in the presence of very low input of virus-derived RNAs or transposons. Also, the spread of primary and secondary siRNAs to distant cells through vascular system provide non-cell autonomous activation of RNA silencing (Melnyk et al., 2011). Different RDR genes exert varying biological functions in plants since *RDR1* and *RDR6* contribute to the accumulation of vsiRNAs in the infected cells (Qi et al., 2009); *RDR2* and *DCL3* are involved in the generation of hc-siRNAs in RdDM pathway thereby controlling the chromatin structure (Willmann et al., 2011); *RDR6* produces dsRNAs from transgenes, endogenous transcripts, and infecting viruses resulting in 21-nt siRNAs targeting respective cognate RNAs (Mourrain et al., 2000; Qu, 2010). *RDR1* has been known to be regulated by stress-related signaling molecules such as salicylic acid (SA), jasmonic acid (JA), ethylene, and nitric oxide, suggestive of its role in systemic acquired resistance (Xie et al., 2001; Qu, 2010; Hunter et al., 2016; Basu et al., 2018). Downregulation of *RDR1* expression in tobacco resulted in systemic spread of potato virus X (PVX) (Xie et al., 2001). *RDR1* favored the generation of 5′-terminal siRNAs of each of the three viral genomic RNAs of cucumber mosaic virus (CMV), whereas vsiRNAs associated with *RDR6* were mapped to the 3′-ends in *rdr1* mutant plants and appears to be *RDR6*-dependent (Wang et al., 2010). The role of dsRNA binding proteins (DRBs) like HYL-1 and DRB-4 is also indispensable for the sRNA-mediated antiviral immunity (Katiyar-Agarwal et al., 2006; Ding, 2010; Jakubiec et al., 2012). Thus, protein components of sRNA-based viral genome silencing are characterized with wide diversity.

Unlike siRNAs, the biogenesis of miRNAs involves the generation of single-stranded RNA with imperfect stem-loop structure called primary miRNA transcripts (pri-miRNA) by the activity of plant RNA polymerase II. The coordinated activity of plant-derived enzymes such as DCL1, DAWDLE, HYPONASTIC LEAVES-1 (HYL1), SERRATE (SE) processes pri-miRNA into mature miRNA duplex (miRNA:miRNA^∗^) *via* precursor miRNA (pre-miRNA) intermediate. The mature miRNA duplex is exported from nucleus to cytoplasm *via* HASTY-1 (HST-1) (Kurihara and Watanabe, 2004; Yu et al., 2008). Either the miRNA or miRNA^∗^ strand regulates gene expression by cleaving the cognate mRNA or by repressing the translation of mRNA based on the extent of complementarity between the miRNA and the mRNA sequences (Doench and Sharp, 2004). Thus, miRNAs differ from siRNAs not only in biogenesis but also in their mode of action.

**FIGURE 1:**
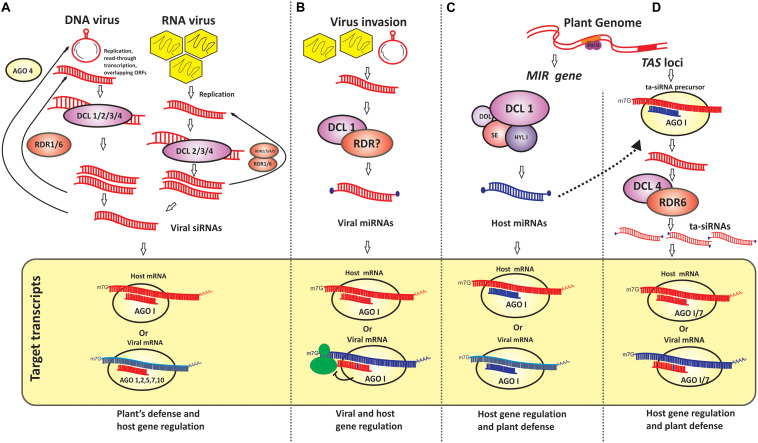
Schematic representation of viral and host-derived small RNAs and their regulatory role **(A)** Viral genome-derived small interfering RNAs (siRNAs) from plant infecting DNA and RNA viruses and their role in regulation of plant gene expression and viral gene downregulation are presented. Host protein components such as dicer-like enzymes (DCL)1 through 4 and argonaute protein (AGO) 1, 2, 5, 7, and 10 participate in this process; **(B)** Viral genome-derived microRNAs (miRNAs) and their regulatory role in modulating the expression of virus genes as it may also function as miRNA mimic to downregulate host mRNA causing the host’s susceptibility. Although little is known about the role of plant proteins in the generation of viral-derived miRNAs this mode of pathogen’s virulence is well recognized in animal-virus interactions; **(C,D)** Host-derived microRNAs and *trans*-acting siRNAs (miRNAs and tasiRNAs) and their role in plant gene regulation and host defense are depicted. Host DCL1 and AGO1 are known in miRNA generation whereas the activities of DCL4, RDR 6, and AGO1, AGO7 are known in tasiRNA mediated gene regulation.

It is increasingly evident that sRNAs, comprising mainly of siRNAs and miRNAs, at the virus–plant interface are the major determinants of an outcome of disease reaction (Palukaitis et al., 2013; Ramesh et al., 2014; Pradhan et al., 2015; Chen et al., 2017) (Figure 1). Additionally, virus-activated siRNAs (vasiRNAs) of 21-nt size are a distinct class of sRNAs that originate from the exons of host genome due to the activity of DCL4 and RDR1. VasiRNAs and host factors [phospholipid flippases, ANTIVIRAL RNAI-DEFECTIVE 2 (AVI2)] are considered to be essential for the conserved anti-viral response in plants (Cao et al., 2014; Guo et al., 2017; Rosas-Diaz et al., 2018). Plants have also evolved sophisticated defense systems such as PAMP-triggered immunity (PTI) and effector-triggered immunity (ETI) (Jones and Dangl, 2006) to counteract other pathogens such as bacteria, fungi, and oomycetes. Plants effectively utilize siRNAs and miRNAs to modulate both the PTI and ETI, and to fine tune the phytohormone pathways and genes involved in pathogenic virulence to the advantage of hosts (Zhang et al., 2011). In this review, we have primarily discussed the role of pathogen-derived sRNAs and their interaction with host mRNAs in shaping the host–pathogen interactions.

## Virus- and Viroid-Derived sRNAs as Pathogenicity Factors

Virus- or viroid genome-derived siRNAs are products of host RNA silencing and hence have been implicated in the defense response of hosts. However, besides VSRs, vsiRNAs have been implicated as pathogenicity factors. Investigations pertaining to the virus or viroid genome-derived siRNAs in the context of host–pathogen interactions revealed that in many instances, the disease symptoms are the consequences of RNA silencing directed against important host genes (Wang et al., 2004). Hairpin RNA expression of potato spindle tuber viroid (PSTVd) genome-derived sequences in tomato recapitulated the characteristic symptoms associated with the natural viroid infection (Wang et al., 2004). Additionally, NGS-based investigations delineating the plant virus–host interactions have characterized a plethora of vsiRNAs, their production hot spots, and sequence variations. Thus, an array of investigations was conducted to ascertain the role of vsiRNAs in virus–host interactions (Table 1). Deep sequencing studies in *Arabidopsis thaliana* infected with tobacco mosaic virus–Cg (TMV-Cg) divulged multiple host genes as potential targets for the vsiRNAs. However, TMV-Cg-derived siRNA-mediated cleavage sites were detected only for two target genes, namely, cleavage and polyadenylation specificity factor (*CPSF30*), and a protein similar to translocon-associated protein alpha (TRAP alpha) using RLM-RACE (Qi et al., 2009). Nonetheless, if the targeted negative regulation of host gene expression is a consequence of fortuitous pairing, or is a bona fide counter–counter defense strategy of the virus to enhance its chances of its own survival remains unresolved.

**TABLE 1:** Interactions between viral and viroid-derived sRNAs and host transcripts causing modulations of host gene expression.

Virus Taxonomy	Genome	Virus–host	Features	References
*Bunyavirales* (order) *Peribunyaviridae* (*Tospovirus*)	ssRNA(+/–)	Tomato spotted wilt virus-*Solanum lycopersicum*	• Interactome of TSWV derived siRNAs and tomato transcripts • Tomato cultivar specific regulation of vsiRNAs	Ramesh et al., 2017
*Bunyavirales* (Order) *Phenuiviridae* (*Tenuivirus*)	ssRNA(–)	Rice stripe virus*-Nicotiana benthamiana*	• Use of VIGS identified eleven genes involved in chlorosis symptoms • Downregulation of *NbeIf4A* is associated with leaf twisting and stunting symptoms	Shi et al., 2016
	ssRNA(–)	Rice stripe virus*-*rice and *Laodelphax striatellus*	• Host-dependent generation and regulation of vsiRNAs	Yang et al., 2018
*Betaflexiviridae* (Sub family *Quinvirinae* and Genus-*Foveavirus*)	ssRNA(+)	Grapevine rupestris stem pitting-associated virus (GRSPaV)- *Vitis vinefera*	• vsiRNAs from both polarity equally represented • U at the 5′-terminus	Miozzi et al., 2013
*Tymoviridae* (*Maculaviridae*)	ssRNA(+)	Grapevine fleck virus (GFkV) – *Vitis vinefera*	• vsiRNAs were of antisense polarity • Major 5′ terminal nt of vsiRNA is G	Miozzi et al., 2013
*Avsunviroidae* (*Pelamoviroid*)	ssRNA	Peach latent mosaic viroid	• Vd-sRNA derived from the pathogenicity determinant region targets host heat shock protein 90	Navarro et al., 2012
*Bromoviridae* (*Cucumovirus*)	ssRNA(+)	Y-satellite of cucumber mosaic virus-*N. benthamiana*	• Target transcript is *N. benthamiana chll* resulting in yellow phenotypes	Shimura et al., 2011; Smith et al., 2011
	ssRNA(+)	Cucumber mosaic virus*- N. tabacum*	• CMV deficient in VSR-2b protein showed multiple putative targets in the host genome	Qiu et al., 2017
*Geminiviridae* (*Begomovirus*)	ssDNA(+/–)	Tomato leaf curl New Delhi virus-*Solanum lycopersicum*	• Short hairpin RNA(shRNA) expression targeting ToLCNDV genome resulted in off-target silencing	Praveen et al., 2010
	ssDNA(+/–)	African cassava mosaic virus (ACMV) and East African cassava mosaic virus-Uganda (EACMV-UG)-Jatropha or cassava	• isomiRs from ACMV and EACMV-UG predicted • Host target genes were involved in biotic response, metabolic pathways and are TFs	Maghuly et al., 2014
	ssDNA(+/–)	Cotton leaf curl Multan virus-*Gossypium hirsutum*	• vsiRNA target host gene encode *MYB* transcription factors. • vsiRNA targeting of host TF promotes viral infection	Wang et al., 2016
	ssDNA(+/–)	Tomato yellow leaf curl virus*-*tomato	• Intergenic region (IR) of TYLCV targets host lncRNA-*SILNR1* only in susceptible cultivar causing stunting and curling leaf traits	Yang et al., 2019
*Luteoviridae* (*Polerovirus*)	ssRNA(+)	Brassica yellows virus	• Differential targeting of respective vsiRNAs	Zhou et al., 2017
*Pospiviroidae*(*Pospiviroid*)	ssRNA	Potato spindle tuber viroid- *Solanum lycopersicum*	• Tomato encoded callose synthase genes *CalS11-like* and *CalS12-like* are silenced by viroid derived Srna	Adkar-Purushothama et al., 2015
	ssRNA	Pospiviroid and *Solanum lycopersicum*	• *SolWD40-repeat* is targeted by planta macho viroid (TPMVd) derived sRNA • Viroid sRNAs and target Arabidopsis genes identified	Avina-Padilla et al., 2015
	ssRNA	Potato spindle tuber viroid- *Solanum lycopersicum*	• Direct interactions between viroid derived sRNAs and tomato mRNAs • Besides viroid sRNAs other factors are also involved in degradation of host mRNAs	Adkar-Purushothama et al., 2017
	ssRNA	Potato spindle tuber viroid*-N. tabacum* and *N. benthamiana*	• amiRNA expression targeting viroid genome silences host gene encoding inorganic pyrophosphatase	Eamens et al., 2014
	ssRNA	Potato spindle tuber viroid*-*tomato	• 3 vd-sRNAs target host receptor serine-threonine kinase (RSTK) • VIGS based expression of vd-sRNAs mimicked infection phenotypes	Adkar-Purushothama and Perreault, 2018
	ssRNA	*Pospiviroids* including *CEVd-*tomato	• *In silico* prediction of host gene targets	Olivier and Bragard, 2018
	ssRNA	Potato spindle tuber viroid*-*tomato	• Tomato *FRIGIDA-like protein 3 (FRL3*) mRNA is targeted causing early flowering	Adkar-Purushothama et al., 2018
	ssRNA	Potato spindle tuber viroid- *Solanum lycopersicum*	• hpRNA expression targeting PSTVd genome resulted in silencing of host genes	Wang et al., 2004
*Potyviridae* (*Potyvirus*)	ssRNA(+)	Turnip mosaic virus-*Arabidopsis thaliana*	• TuMV genome encoded miRNAs (TuMV-mir-S1 and TuMV-mir-S2) • HVA22D gene in Arabidopsis is the targeted	Shazia, 2010
	ssRNA(+)	Sugarcane mosaic virus- *Zea mays* L.	• Differential target potential of vsiRNAs from viral genome strandedness • Multiple pathways of host transcriptome targeted	Xia et al., 2014
	ssRNA (+)	Potato virus-Y (strains PVY-N, PVY-NTN and PVY-O) - *Solanum tuberosum*	• Global effects of PVY strain derived vsiRNAs on potato transcriptome • vsiRNAs target genes involved in hormone signaling, plant defense and stress response	Moyo et al., 2017
	ssRNA(+)	Papaya leaf distortion mosaic virus-Papaya	• A total of 23,591 putative targets sites in papaya mRNAs	Zhao et al., 2018
*Potyviridae* (*Poacevirus*)	ssRNA(+)	Sugarcane streak mosaic virus- *Saccharum officinarum*	• SCSMV derived miRNA pre-miR-16 and regulation of plastidial isoprenoid biosynthesis genes.	Viswanathan et al., 2013
	ssRNA(+)	Banana bract mosaic virus-banana	• Existence of BBrMV-derived miRNA2 in infected leaves	Sankaranarayanan et al., 2020
*Reoviridae* (Sub family-*Spinareovirinae*, Genus: *Fijivirus*)	dsRNA	Southern rice black-streaked dwarf virus*-*rice	• Potential rice target genes (844) including resistance or PR genes and host RNA silencing factors [*OsDCL2a* and *OsAGO17*] • Molecular basis of barren grain in SRBSDV infection	Xu and Zhou, 2017
*Tombusviridae* (*Betacarmovirus*)	ssRNA(+)	Hibiscus chlorotic ring spot virus -*Hibiscus cannabinus*	• Entry of HCRSV RNA into nuclei • Predicted viral miRNA (hcrsv-miR-H1-5p) shown to downregulated viral gene expression	Gao et al., 2012
*Tombusviridae* (*Umbravirus*)	ssRNA(+)	Pea enation mosaic virus 2- *N. benthamiana*	• Differential targeting of respective vsiRNAs	Zhou et al., 2017
*Virgaviridae* (*Tobamovirus*)	ssRNA(+)	Tobacco mosaic virus Cg-*Arabidopsis thaliana*	• Target transcripts are *At1g30460* and *At2g16595*	Qi et al., 2009
	ssRNA(+)	Cucumber green mottle mosaic virus-Cucumber	• Potential host gene targets (92 genes) were identified • Predominant vsiRNAs of 21–22 nt in length	Li et al., 2016
	ssRNA(+)	Tobacco mosaic virus*-N. benthamiana*	• TMV-vsiRNA 22 nt-derived from its 3′UTR • vsiRNA targets C2-domain abscisic acid (ABA)-related (CAR) 7-like protein which regulates ABA responsive genes	Guo and Wong, 2020
*Virgaviridae (Furovirus)*	ssRNA(+)	Chinese wheat mosaic virus−*Triticum aestivum*	• CWMV-derived vsiRNA-20 targets vacuolar (H+)−PPases (VPs)	Yang et al., 2020

*TSWV, tomato spotted wilt virus; VIGS, virus induced gene silencing; siRNAs, small interfering RNAs; Vd-sRNA, viroid-derived sRNA; CMV, cucumber mosaic virus; VSRs, viral suppressors of RNA silencing; ToLCNDV, tomato leaf curl New Delhi virus; TFs, transcription factors; vsiRNAs, virus-derived RNAs; TYLCV, tomato yellow leaf curl virus; lncRNA, long non-coding RNA; VIGS, virus induced gene silencing; PVY, potato virus Y strains; SCSMV, sugarcane streak mosaic virus; BBrMV, banana bract mosaic virus; SRBSDV, Southern rice black-streaked dwarf virus; HCRSV, hibiscus chlorotic ring spot virus; TMV, tobacco mosaic virus; ABA, abscisic acid; CAR, C2-domain ABA-related; CWMV, Chinese wheat mosaic virus.*

Along the similar lines, a biologically relevant question of host-specific symptom expression was investigated in CMV-Y satellite RNA (Y sat) model. It was known that yellow mosaic symptom appears when CMV-Y sat infects *Nicotiana tabacum*, whereas these characteristic symptoms were lacking when the virus infects either Arabidopsis or tomato. Unraveling the molecular mechanism underlying this host-specific phenomenon established that CMV-Y sat RNA-derived siRNAs downregulated the host protoporphyrin chelatase subunit I gene (*chll*) involved in chlorophyll biosynthesis resulting in yellow symptoms. Corroboratively, expression of RNA silencing constructs targeting Y sat RNA also caused yellowing symptoms. Moreover, incorporation of RNA silencing gene constructs with the *chll* sequence of Arabidopsis and tomato manifested typical yellow mosaic symptoms in an otherwise non-responsive host. Thus, this study demonstrated the role of sub-viral agent and associated vsiRNAs in inducing characteristic symptoms in the natural host (Shimura et al., 2011). Furthermore, the direct role of CMV Y-Satellite RNA (Y-Sat RNA)-derived siRNA in producing characteristic yellow symptoms as a consequence of vsiRNA targeting chlorophyll biosynthesis gene *CHLI* was documented (Smith et al., 2011). CMV-2b-deficient mutant infection of *N. tabacum* generates vsiRNAs predominantly from the viral sense strand, which had many putative targets in the genome of host affecting various cellular and biological processes (Qiu et al., 2017).

Chlorotic symptoms associated with many viral infections are presumed to be the result of downregulation of genes of chloroplastic and photosynthetic machinery. Satoh et al. (2010) showed that chloroplast-associated genes of rice are downregulated during rice stripe virus (RSV) infection causing leaf chlorosis. The molecular basis of this interaction was elucidated by Shi et al. (2016) utilizing virus-induced gene silencing (VIGS) system in RSV-*N. benthamiana* model. Among the differentially expressed genes, 11 (nine of them were chloroplastic genes) were found to be involved in inducing chlorosis. Also, the eukaryotic translation initiation factor 4A (*NbeIf4A*) was downregulated resulting in leaf twisting and stunting symptoms associated in RSV infection. Interestingly, vsiRNA-4A derived from RSV showed sequence complementarity with *NbeIf4A* mRNA, and artificial miRNA expression of these vsiRNAs demonstrated silencing of *NbeIf4A* gene (Shi et al., 2016). Further, 5′ RACE analysis proved that vsiRNA-4A cleaves target mRNA at a position upstream of vsiRNA binding site similar to CMV satellite-derived small RNA (satsiR-12) targeting of 3′ UTR of CMV RNA (Zhu et al., 2011) suggesting the non-canonical processing of target mRNAs by vsiRNAs. In Southern rice black-streaked dwarf virus (SRBSDV) infected rice, vsiRNAs potentially target host genes of chloroplastic origin, and *AGO17* gene involved in pollen development suggesting the role of vsiRNAs in leaf-associated disease symptoms, and barren grains caused due to SRBSDV (Xu and Zhou, 2017). Later, Yang et al. (2019) proved the role of siRNAs derived from the intergenic region (IR) of tomato yellow leaf curl virus (TYLCV) in symptom development in tomato. The IR has a 25-nt sequence, perfectly complementary to a long non-coding RNA (lncRNA, *SILNR1*) in susceptible tomato cultivars. However, this sequence complementarity is lacking in the resistant plants due to the 14 nt deletion in the host genome. The viral siRNAs derived from the IR have the ability to silence the *SILNR1* in susceptible cultivars actuating the stunting and curling leaf traits. Further, *SILNR1* overexpressing lines showed reduced accumulation of TYLCV suggestive of the negative crosstalk between the vsiRNAs and the lncRNAs (Yang et al., 2019).

Viroids are also the inducers and targets of RNA silencing owing to intramolecular base pairing of their genomic RNAs and replication intermediate-dsRNAs (Pallás et al., 2012). RNA silencing of viroid RNAs and accumulation of vd-sRNAs during viroid infections suggested that these sRNAs potentially target host mRNAs to induce the disease symptoms (Tsushima et al., 2015). Chloroplast replicating peach latent mosaic viroid specifically targets host mRNA encoding heat shock protein 90 (*HSP 90*), causing characteristic symptoms and suppressing the host defense network (Navarro et al., 2012). Earlier, the significance of vd-sRNAs in inducing the symptoms was demonstrated in HSVd-infected *N. benthamiana* plants deficient in *RDR6* activity (Gómez et al., 2008). Artificial microRNA (amiRNA)-based silencing of a virulence-modulating region of PSTVd genome inadvertently downregulated the host soluble inorganic pyrophosphatase gene. The silencing of inorganic pyrophosphatase genes of *N. tabacum* and *N. benthamiana* caused phenotypic features that mimicked the typical viroid disease symptoms, clearly suggesting the off-target effects of vd-siRNA in targeted host genes (Eamens et al., 2014). Contrarily, pelargonium line pattern virus (PLPV) (family *Tombusviridae*) infecting *N. benthamiana* neither elicit the host DCL induction nor the RDR6 activity to mount antiviral defense; however, PLPV vsiRNAs, representing the entire genome of the virus, are abundant in the non-symptomatic plants. Despite the abundance of vsiRNAs, accumulation of the host mRNAs is not affected. Infection caused alterations in host miRNA concentrations; nonetheless, it is not correlated with any phenotypic abnormalities. Hence, it is pertinent to investigate other host factors that remain uncharacterized yet but could be associated with viral pathogenesis and symptom appearance (Pérez-Cañamás et al., 2017).

## Virus- and Viroid-Derived sRNAs Attenuate Host Defense System

Genome-wide target genes of *Vitis vinifera* for vsiRNAs derived from grapevine fleck virus and grapevine rupestris stem pitting-associated virus were identified (Miozzi et al., 2013). The uncapped host transcripts in the degradome library, especially the transcripts encoding ribosomal subunits, and others involved in ribosome biogenesis compelled Miozzi et al. (2013) to propose the vsiRNA-mediated cleavage of transcripts. Among the identified target transcripts were those that encode for stress-associated host proteins suggesting the plausibility of compromised stress responsiveness. vsiRNAs derived from cucumber green mottle mosaic virus (CGMMV) exhibited sequence complementarity to 92 host genes, and these potential target genes are involved in crucial functions such as maintenance of cellular structure, cellular processes, localization, sugar metabolic pathways, and response to stimuli suggesting the versatility of CGMMV-derived vsiRNAs in modulating the host defense regulatory systems (Li et al., 2016). Similarly, vsiRNAs derived from CGMMV*-*infected watermelon had potential target sites in host pathways including ribosome biogenesis, RNA biology, and target genes vary from multiple transcription factors (TFs) to nucleotide binding site–leucine-rich repeats (NBS-LRR) involved in host defense pathways (Sun et al., 2017). Deep sequencing investigations of SRBSDV-infected rice plants showed that 844 rice genes were potential targets for the most abundant vsiRNAs. The target rice genes include resistance or pathogenesis-related genes coding for F-box/LRR proteins and receptor-like protein (RLP) kinase, stress proteins, pathogenesis, and developmental aspects of the host and which were confirmed by qRT-PCR. The host RNA silencing factors such as *OsDCL2a* and *OsAGO17* are also targeted by the vsiRNAs derived from SRBSDV (Xu and Zhou, 2017). Therefore, the target genes such as NBS-LRR, RLP-kinase, DCLs, and AGO signify the effects of vsiRNAs on host defense systems.

**FIGURE 2:**
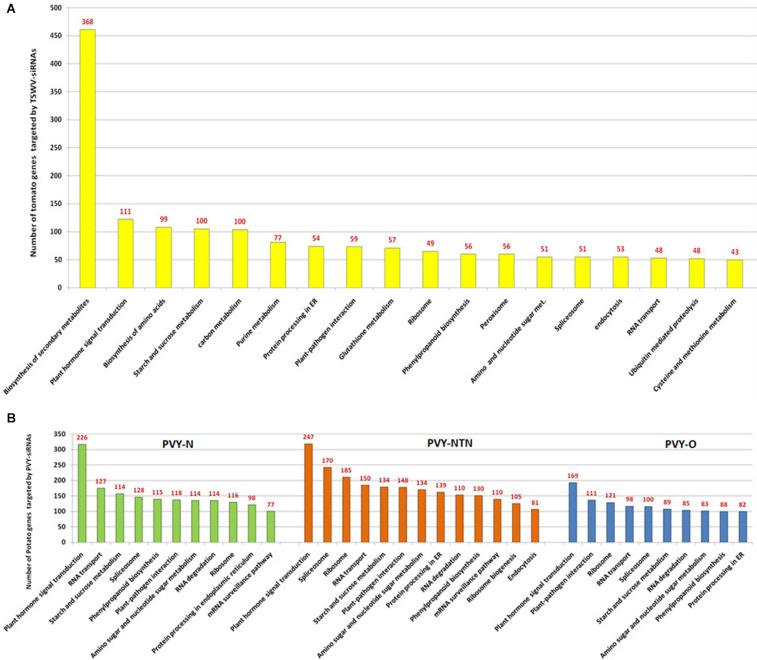
Interaction of siRNAs derived from viruses (tomato spotted wilt virus [TSWV] and potato virus Y [PVY]) infecting solanaceous crops and their respective host transcriptome. **(A)** Potential impact of tomato spotted wilt virus (TSWV)- derived siRNAs (vsiRNAs) on the diverse genes of host tomato involved in wide repertoire of various metabolic pathways. Tomato metabolic pathways with more than 50 hits alone are depicted and the numbers of TSWV-derived siRNAs affecting each pathway are depicted above the bars (Adapted from Ramesh et al., 2017). **(B)** Potential impact of potato virus Y (PVY)-derived genomic siRNAs on the diverse genes of host potato encoding enzymes and proteins involved in various metabolic pathways. Potato metabolic pathways with more than 50 hits alone are depicted along with the numbers of PVY-derived siRNAs affecting each pathway presented above the bars (Adapted from Moyo et al., 2017).

Analysis of abundant class of tomato spotted wilt virus (TSWV)-derived siRNAs from infected tomato identified that 2,450 unique vsiRNAs have potential to interact with tomato transcriptome. It is noteworthy that tomato cultivars exhibited differential molecular response to the TSWV-derived vsiRNAs that potentially targeted the host mRNAs. Tomato cultivar (Red Defender) containing the virus resistance-conferring loci (+*Sw-5*) has the ability to overcome the vsiRNA-mediated targeting of NBS-LRR class R-genes. The wide range of host mRNA targets indicate that vsiRNAs are not mere pathogenicity factors but also are the determinants of the outcome of host–virus interaction (Ramesh et al., 2017) (Figure 2A). vsiRNAs derived from the genomic components of three different strains of potato virus Y: N, NTN, and O and the prospect of vsiRNA:host mRNA interaction was investigated (Moyo et al., 2017) (Figure 2B). The number of potential target sites on the transcriptome of potato for vsiRNAs varied with the potato virus Y strains. vsiRNAs of PVY-NTN exhibited more propensities for targeting potato transcriptome than that of PVY-N and PVY-O, both of which showed comparable levels of targets (Moyo et al., 2017). Thus, PVY–potato interactions at the sRNA interface proved that PVY–siRNAs not only act as pathogenicity determinants but also potentially suppress the host defense systems and other cellular developmental activities (Moyo et al., 2017). RSV genome-derived sRNAs and mRNAs of host rice and insect vector brown planthopper (*Laodelphax striatellus*) were investigated to gain insights about the regulatory roles of vsiRNAs in different hosts. Among the downregulated genes of RSV-infected rice, 70% of the genes had potential vsiRNA target sites, and these genes were found to be enriched in carbohydrate binding and kinase activity. Similarly, 47.5% of downregulated genes in RSV-infected plant hoppers were potential targets for vsiRNAs and were involved in activities such as cuticle formation, peptidase, and oxido-reductase. vsiRNA profiles of the same virus instigate different molecular responses depending on the host system reflecting diverse biological requirements (Yang et al., 2018). Thus, the preferential targeting of defense-related genes of both the rice and plant hoppers by vsiRNAs could be considered as a counter-offensive strategy employed by the virus to improve its own survival chance. The target mRNAs of common vsiRNAs showed differences not only in sequence complementarity with vsiRNAs but also in target positions and secondary structural characteristics adding one more facet to the layer of gene regulation (Pooggin, 2018).

Viroid-derived sRNA (vd-sRNA) from the virulence modulating genomic region of PSTVd specifically targets multiple callose synthase genes of the host (*CalS11-like* and *CalS12-like*) involved in callose deposition in pollen mother cell walls and during pathogen infection (Adkar-Purushothama et al., 2015). The importance of vd-sRNA in modulating host defense genes such as *CalS11-like* and *CalS12-like* has been further reinforced in viroid genome swapping experiments. Also, the sequence polymorphism in *CalS11-like* mRNA in tomato cultivars influenced viroid accumulation, suggesting the influence of vd-sRNAs in manifesting disease reaction. Comparative genomic analysis revealed a set of potential target genes in Arabidopsis genome for the sRNAs derived from 29 species of viroids belonging to the family *Pospiviroidae*. A conserved *SolWD40-repeat* gene of tomato has been validated as a target gene for vd-sRNA (Avina-Padilla et al., 2015). The plant WD-40 repeat proteins have been reported to play a role in histone modifications, signal transduction pathways (Mascheretti et al., 2013), and also in abiotic stress tolerance (Kong et al., 2014). However, the biological significance of the downregulation of WD40 during viroidal infection is still unknown. PSTVd–tomato interactions have further uncovered the effect of vd-sRNAs of both the PSTVd (+) and (-) strands on the tomato transcriptome. The potential vd-sRNA target tomato genes *viz*., 3′ UTR of the *chloride channel protein CLC-b-like* and *RPS3a* are downregulated. However, parallel analysis of RNA ends show that target mRNA is cleaved at sites other than predicted by *in silico* studies indicating the vd-sRNA-induced independent degradation of host mRNAs (Adkar-Purushothama et al., 2017).

Understanding the interaction between *PSTVd* genome and defense-related genes of tomato identified siRNA production hot spots in viroid genome. Of all the vd-sRNAs, 3 vd-sRNAs that target different transmembrane receptors such as receptor serine–threonine kinase (RSTK) involved in host defense process were identified. Transient expression of all the three vd-siRNAs using a VIGS model based on osa-MIR528 backbone mimicked viroid infection phenotypes (Adkar-Purushothama and Perreault, 2018).

## Virus- and Viroid-Derived sRNAs Enhance the Virulence

vsiRNAs from the antisense strand of sugarcane mosaic virus (SCMV) exhibited multiple target sites in the maize transcriptome compared to the siRNAs from the sense strand denoting the strand-specific role of vsiRNAs in host gene regulatory pathways. Furthermore, a broad range of host transcriptome targets involving various pathways such as gene regulation, energy metabolism, cellular defense, and plant signaling underlines the predominance of vsiRNAs in enhancing the virulence of pathogen (Xia et al., 2014). Cotton leaf curl Multan virus (CLCuMuV) and the associated betasatellite cotton leaf curl Multan betasatellite (CLCuMuB)-derived sRNAs (1,723 vsiRNAs) have 2,462 potential target sites in the cotton transcriptome, and most common putative targets are TFs. VIGS-based silencing of putative host *MYB* TF caused enhanced (>threefold) CLCuD viral DNA accumulation (Wang et al., 2016). Though high viral nucleic acid levels do not necessarily translate into an enhanced virulence, the concentration and activities of viral proteins are major virulence-enhancing factors (Culver and Padmanabhan, 2007). Thus, this work proved the involvement of vsiRNA targets in response to CLCuD infection, and further studies are warranted to decipher the biological implications of this interaction in CLCuD regulatory network.

Dual infection of maize chlorotic mottle virus (MCMV) and SCMV in maize revealed possible roles of vsiRNAs in synergistic infection (Xia et al., 2016). In SCMV-infected maize plants, equal distribution of vsiRNAs from positive- and negative-sense strands of the virus was documented, whereas in MCMV-infected plants, vsiRNAs originated mainly from the viral genomic RNAs. Nevertheless, the vsiRNAs showed little difference in the putatively targeted host pathways, as “metabolic pathways” followed by “biosynthesis of secondary metabolites” were abundantly enriched as targets (Xia et al., 2016). However, vsiRNA profile of *N. benthamiana* plants co-infected with a polerovirus, brassica yellows virus (BrYV), and an umbravirus, pea enation mosaic virus 2 (PEMV 2), showed differences in their target pathways enhancing the synergistic infection and virulence (Zhou et al., 2017). Similarly, analysis of vsiRNA-target genes during the co-infection of odontoglossum ringspot virus and cymbidium mosaic virus point toward the syngergistic interaction (Pai et al., 2020). Chinese wheat mosaic virus (CWMV)-derived vsiRNA-20 has been shown to regulate wheat vacuolar (H+)-PPases (VPs) thereby suppressing the cell death creating a relatively favorable environment for the survival of CWMV (Yang et al., 2020). It establishes that vsiRNA modulate the host physiological conditions by altering the cellular pH conditions suitable for CWMV replication (Yang et al., 2020).

Viroid genomic RNAs are lncRNAs (Shimura and Masuta, 2016) that subvert and reprogram the host epigenetic RNA silencing to intensify the viroid replication. Physical interaction of HSVd with the host Histone Deacetylase 6 (*HDA6*) led to the postulate that favorable transcriptional changes are induced in the viroid infected cells so as to recognize the pathogenic RNA as an RNA pol II template so that viroid replication is enhanced (Castellano et al., 2016). Infectious RNAs such as viroids and some RNA viruses cause diseases and elicit the activation of host RNA silencing pathway. Comprehensive genome-wide responses of tomato upon PSTVd infection evinced expression of host lncRNAs, alternative-splicing of host proteins, modulations in host-miRNA mediated cleavage of transcripts, induction of phased siRNAs (phasiRNAs), and basal defense related genes. This study had put forth a model depicting co-operative action between RNA silencing and host basal defense system is crucial to avert the spread of nuclear replicating viroids such as PSTVd and others (Zheng et al., 2017). Additionally, alterations in phytohormone signaling pathways and metabolic pathways were observed when citrus bark cracking viroid (CBCVd) infects *Humulus lupulus* L. (Mishra et al., 2018). Computationally predicted vd-sRNAs from eight pospiviroids including CEVd were mapped to tomato CDS and hypothetical PTGS targets were identified. Analysis of target genes revealed that the gene ontology terms such as cytoskeletons, membrane transporters and kinases were over represented (Olivier and Bragard, 2018). Five genes such as translational activator *gcn1*, argonaute 2A, epoxide hydrolase and putative RNA-binding proteins were found to be targets based on *in silico* analysis by performing parallel analysis of RNA ends (PARE) (Olivier and Bragard, 2018). These genes could potentially alter the fitness of CEVd or influence the symptoms of viroid infection.

## Virus- and Viroid-Derived sRNAs Perturb Normal Host Development

Induction of early flowering in plants infected with viroids is well-known (Hosokawa et al., 2004). Intriguingly, the molecular role of lncRNAs in affecting flowering in plants was not known until recently. Viroid genomic RNAs share characteristic features of lncRNAs (Shimura and Masuta, 2016). PSTVd infection attenuates the expression of tomato mRNA coding for FRIGIDA-like protein 3 (FRL3) by a vd-siRNA leading to early flowering. Vd-siRNAs from the left terminal genomic region were shown to target *FRL3* mRNA by PARE sequencing and RLM-RACE techniques. Thus, it provided the direct evidence for the role of a vd-siRNA in modulating the expression of host-mRNA wherein, vd-siRNA not only downregulated *FRL3* mRNA but also induced early flowering in tomato (Adkar-Purushothama et al., 2018). Corroborating this observation, tomato planta macho viroid (TPMVd) and Mexican papita viroid (MPVd) infection caused downregulation of flower-specific *SlOVA6*, while expression of *SlBIGPETAL1* was upregulated thus, the genetic elements involved in the fruit and flower development were modulated during viroid infection. Nonetheless, the function of vd-siRNAs in this downregulation has not been explored (Aviña-Padilla et al., 2018). These research findings pertaining to vd-siRNA-induced changes in host transcriptome provide clues to the regulatory changes in the reproductive gene expression of tomato. Tomato mosaic virus (TMV)-derived vsiRNA originating from its 3′ UTR has been shown to target C2-domain abscisic acid (ABA)-related (CAR) 7-like protein of *N. benthamiana*. Interestingly, *Nb-CAR7* expression level is negatively correlated with the TMV RNA accumulation. Also, *Nb-CAR7* mediates its antiviral role by modulating the expression of host abscisic acid responsive genes (Guo and Wong, 2020).

## Virus-Derived miRnas and Host Gene Regulation

The small RNA effector molecules are diverse in nature hence, besides siRNAs another major class of endogenous small non-coding RNAs called microRNAs (miRNAs) play main role in growth and development of organisms. Animal-infecting DNA viruses encode miRNAs that modulate the expression of host genes and to regulate self gene expression (Sullivan and Ganem, 2005; Skalsky and Cullen, 2010; Kincaid et al., 2012). miRNAs of plant infecting viruses have been identified in few instances. The finding of turnip mosaic virus (TuMV)-derived sRNAs in *Arabidopsis* nuclei led to the computational prediction and experimental validation of miRNA generating loci in TuMV genome (Shazia, 2010). TuMV-derived miRNAs, namely, TuMV-mir-S1 and TuMV-mir-S2 were found to downregulate the host stress-responsive gene HVA22D in *Arabidopsis*, which is crucial for cellular defense pathway and regulated by abscisic acid (Shazia, 2010). Similarly, single-stranded positive sense RNA virus hibiscus chlorotic ring spot virus*–*(HCRSV) was shown to enter plant nuclei and viral genome-derived miRNA (hcrsv-miR-H1-5p) was identified to be involved in the regulation of viral replication (Gao et al., 2012). In another instance, miRNAs encoding potential of sugarcane streak mosaic virus (SCSMV) belonging to family *Potyviridae* was revealed where the viral miRNA generating loci were validated through stem-loop RT-qPCR, and the host target genes were predicted (Viswanathan et al., 2013).

Computational analysis of SCSMV also revealed the hairpin-RNA structured miRNA precursor in its genome. The predicted miRNA precursor, pre-miR-16, was present in the 3′UTR region of the viral genome and its expression was demonstrated. Furthermore, the reduced chlorophyll and carotenoids content in SCSMV-infected leaves was co-related with the virus-derived miRNA-mediated silencing of plastidial isoprenoid biosynthesis pathway (Viswanathan et al., 2013). Recently, miRNAs encoded by banana bract mosaic virus (BBrMV) was explored, and the predicted miRNA2 was found to be conserved in all the BBrMV isolates investigated, and many of its target transcripts play a role in virus infectious cycle (Sankaranarayanan et al., 2020).

These studies thus unravel hitherto an unknown mode of counter-defense strategy employed by the plant-infecting viruses. Plant virus-derived miRNAs is a rarity when compared to miRNAs of animal viruses (Kincaid and Sullivan, 2012). Viruses infecting animals employ strategies like latency or persistent infection and regulate mRNA expression through translational repression; hence, miRNAs are the preferred effector molecules to achieve these gene regulatory effects. Interestingly, functional characterization of the animal virus-derived miRNAs revealed their role in evading host immune response (Dölken et al., 2010) and protracting the survival of infected cells (Xu et al., 2011).

The entry of viral nucleic acid into the nucleus is pertinent for the miRNA production as miRNA-processing proteins are nuclear specific. Since majority of the plant-infecting viruses are RNA viruses that seldom enters nucleus, it is speculated that miRNAs derived from plant viruses are uncommon. However, geminiviruses having DNA genomes enter the nucleus of a plant cell, hence, are the potential candidates for the search of viral genome-derived miRNAs. Search for miRNA pre-hairpin-like sequences in 39 begomoviruses (family *Geminiviridae*) infecting *Cassava* and *Jatropha* revealed nine pre-miRNAs from the genomes of African cassava mosaic virus (ACMV) and five pre-miRNAs from East African cassava mosaic virus- Uganda (EACMV-UG). In addition, DNA A-derived ORFs AC1, AC2, AC4, and IR regions of the begomoviruses exhibited miRNA-coding potential. Viral -derived miRNAs were also validated through stem-loop RT-PCR (Maghuly et al., 2014). Thus, the presence of miRNA coding potential and functional miRNAs in the genomes of one of the severely infecting plant viruses further emphasize the diverse role of sRNAs in plant virus interactions.

## Viral Suppressors vs. vsiRNAs

Virus infections cause a broad range of symptoms in the host plants. The molecular basis of the symptom induction could partly be ascribed to the virus genome-encoded suppressor proteins of RNA silencing. Evolutionarily, viral suppressors of RNA silencing (VSRs) are considered to be of recent origin and exhibit wide nucleotide sequence diversity across the virus species. VSRs interfere in all the crucial steps of RNA silencing right from the recognition of viral RNA (Mérai et al., 2005), dsRNA dicing (Mérai et al., 2006), assembly of RISC (Lakatos et al., 2006; Zhang et al., 2006; Bortolamiol et al., 2007), through RNA targeting and amplification of silencing signals so as to attenuate the host defense responses. These VSRs exhibit high affinity for sRNAs than HEN1 methyltransferase, thereby negating the 2-O′ methylation of sRNAs (Ebhardt et al., 2005). Also, some viral suppressors are characterized with glycine/tryptophan (GW/WG) repeat motifs mimicking the host proteins that interact with AGO. In this manner suppressor proteins not only affect sRNA loading but also alter the pool of Dicers in the cellular environment and thereby severely impair the AGO-dependent homeostatic pathway (Azevedo et al., 2010). Alternatively, P19 modulates the levels of AGO1 protein by inducing the expression of host miRNA168 (Varallyay et al., 2010). Host RDRs that are involved in amplification of silencing signals are also targeted by the viral suppressors. For instance, V2 of the TYLCV interacts with suppressor of gene silencing-3 (SGS3) that mediates RDR6-based amplification of silencing signals (Glick et al., 2008). Similarly, turnip mosaic virus encoded suppressor protein, VPg, orchestrates the degradation of SGS3 and its interacting and functional partner RDR6 through 26S ubiquitin-proteasome and autophagy pathways (Cheng and Wang, 2017). Suppressors proteins of viruses belonging to family *Geminiviridae* modulate host factors involved in epigenetic modification of viral genomes (Yang et al., 2011). Thus, VSRs form the major back-bone of counter-defense strategy of the viruses.

The sRNA sequestration capability of VSRs is considered to be a potent strategy because siRNA duplexes are powerful silencing signals that can travel faster than virion particles to the uninfected cells (Csorba and Burgyán, 2016). Hence, VSRs that bind siRNAs could profoundly compromise the antiviral capability of the host. Thus, a crucial factor that could potentially diminish the vsiRNA activities under *in vivo* conditions is the prevalence of VSRs. Many VSRs possess sRNA sequestration capability so that it bind siRNA duplexes or miRNA:miRNA^∗^ thereby limiting the active pool of siRNAs in effecting trans-silencing of host transcripts (Kasschau et al., 2003; Chapman et al., 2004; Lakatos et al., 2006).

It is also known that interference of suppressor proteins in RNA silencing pathways results in the manifestation of disease symptoms. Although, ectopic expression of viral suppressors in plants has recapitulated the characteristic symptoms of the viral diseases, time, and space dimensions of the symptoms expression do not mimic the natural infection warranting utmost care in attributing the disease symptoms only to suppressors. Additionally the pathological manifestations of symptoms are attributed to the competitions arising due to the interplay between the host developmental pathways and antiviral defense pathways (Pallas and García, 2011). The symptomatic phenotypes could be due to suicidal responses of plants causing hypersensitive reaction (HR), effect of viral and plant factors in endogenous hormonal regulatory pathways (Alazem and Lin, 2015; Vinutha et al., 2020), interfering with the host-derived cell cycle regulatory proteins, and the role of viral non-coding regions (Pallas and García, 2011). Also, symptoms due to viral infection are influenced by the environmental factors such as temperature, light intensity, water status, and atmospheric CO_2_ concentration. At temperatures higher than the critical limit, plants accumulate high concentration of viral RNA molecules and it is correlated with symptom development (Szittya et al., 2010). Similarly, high light intensity does not affect the localized RNA silencing; however, the spread of secondary signals of RNA silencing are inhibited due to the light-induced changes in source–sink relationship (Patil and Fauquet, 2015). Nevertheless, in depth molecular studies delineating the role of VSRs and vsiRNAs during these environmental extremities, which in turn determine symptom appearance remains elusive. In this context, vsiRNAs are the most likely candidates involved in symptoms induction because expression of specific vsiRNA/vd-siRNA has been correlated with the appearance of characteristic disease symptoms (Shimura et al., 2011; Smith et al., 2011; Eamens et al., 2014; Shi et al., 2016). Although reports showed how vsiRNAs modulate the expression of host transcripts, a fundamental question as to how vsiRNAs escape the activity of VSRs remains unclear. Thus, the idea that a fraction of vsiRNAs functionally target host genes is at odds since most viruses encode functionally active VSRs that suppress RNA silencing. It warrants spatio-temporal separation of viral suppressor expression and vsiRNA-targeting of host mRNAs. At this juncture, the phenomenon of symptom recovery, characterized with remission of viral disease symptoms, could provide clues as it is also accompanied by reduced concentrations of viral proteins below a threshold level. Recent evidences indicate that symptom recovery phenomenon during viral infection can occur not only against viruses encoding weak suppressor proteins but also against a strong viral suppressor of RNA silencing when the host RNA silencing and intercellular molecular communication overpowers the activity of viral suppressors. Thus, symptom recovery involves concerted action of PTGS defense pathway for robust production of 21–22-nt size class secondary vsiRNAs and TGS pathway which is known to facilitate non-cell-autonomous silencing signaling (Kørner et al., 2018).

In consequence, plant parts exhibiting recovery phenomenon are suitable models to study the relative effects of viral suppressors and viral-derived siRNAs in defining the remission of symptoms. Instances of symptom recovery characterized with reduced viral proteins such as VSRs accompanied with high levels of viral nucleic acids were documented (Jovel et al., 2007; Ghoshal and Sanfaçon, 2015; Nie and Molen, 2015). Thus, it appears that the trade-off between the VSRs and vsiRNAs to achieve their respective threshold determines the phenomenon of symptom recovery. These conditions could potentially swing the tug of war in favor of the host by producing more vsiRNAs to target viral mRNAs. However, vsiRNA population that outweighs VSRs within the cellular pool could simultaneously target either the viral or host RNAs. Viruses deploy self-attenuation mechanism to achieve this balance. For example, the suppressor function of 2b protein is attenuated by the coat protein (CP) of CMV and this interaction between CP and viral nucleic acids enhances the production of vsiRNAs (Zhang et al., 2017). Furthermore, the most abundant class of vsiRNAs in plants constitutes secondary vsiRNAs, which are not the derivatives of viral replicative intermediates. Thus, VSRs that are not effective in antagonizing the activity of host factors such as RDRs, and SGS3 that are involved in secondary vsiRNA production provide another escape route for vsiRNAs from the clutches of VSRs. It is also becoming clear that VSRs are versatile and they dynamically integrate multiple activities such as suppression of RNA silencing, protein-based immunity, sub-cellular organization, and co-ordination of non-silencing activities such as movement, protease, replicase, and coat protein. When the cellular demand diverts the VSRs for non-silencing activities, vsiRNAs are free in the cellular pool. Furthermore, the emerging novel host factors (antiviral RNA1-defective2 or AVI2) were shown to have a role in the biogenesis of highly abundant vsiRNAs, and virus-activated siRNAs (vasiRNAs), which could shift the balance in favor of vsiRNAs over VSRs (Guo et al., 2018).

vsiRNAs that escape the binding of suppressor protein could potentially act on the host transcripts. Nonetheless, the silencing activity of vsiRNAs *in vivo* is determined by many factors. For instance, VSRs differentially inhibit various classes of sRNAs as Tombusviral P19 effectively binds vsiRNAs over host miR159; hence, the pathogenicity effect of vsiRNAs are less pronounced. Also, sub-cellular localization of suppressor proteins determines their sRNA binding efficiency since nuclear localized suppressor (e.g., TEV encoded Hc-Pro) efficiently affects host miRNA and siRNA pathways. Correspondingly, sub-cellular localization of a 126-kDa replicase protein of severe and mild isolates of pepper mild mottle virus (PMMoV) has been attributed with differential symptoms expression in *N. benthamiana*. Although, the authors reasoned that interaction of 126 kDa protein with unknown host factors for differences in the intensity of symptoms, differential impact of the proteins on the PMMoV-derived siRNAs cannot be ruled out. Moreover, suppressors enhance the virulence of viral pathogens by aiding in the accumulation of viral nucleic acids. Nonetheless, as already stated, viral genomic RNA titers do not always correlate with the symptom severity even in the presence of a strong or a weak suppressor protein (Aguilar et al., 2015).

## Target Host–Genes and Implications

The repertoire of host genes functionally targeted by vsiRNAs shows that the characteristic disease symptoms are produced when the host genes involved in chlorophyll biosynthesis and photosynthetic machinery are targeted (Satoh et al., 2010; Shimura et al., 2011; Smith et al., 2011; Shi et al., 2016; Xu and Zhou, 2017). Alternatively, developmental or morphological defects such as leaf twisting and curling, barren grains, and early flowering are induced when the constitutively expressed host genes are targeted by vsiRNAs (Shi et al., 2016; Xu and Zhou, 2017; Adkar-Purushothama et al., 2018; Yang et al., 2019). Such effects of vsiRNAs on the host transcriptome modulations resulting in leaf morphogenesis and chlorophyll biosynthesis could be advantageous to the pathogens as it attracts insect vectors for effective transmission. Nevertheless, vsiRNAs specifically target the host defense genes such as NBS-LRR (Sun et al., 2017), resistance or pathogenesis-related genes coding for F-box/LRR proteins and RLP kinases, receptor serine-threonine kinase (RSTK) (Adkar-Purushothama and Perreault, 2018), stress proteins (Miozzi et al., 2013), heat shock proteins (Navarro et al., 2012), and MYB TFs (Wang et al., 2016). Additionally, the host RNA silencing factors such as *OsDCL*2a and *OsAGO*17 are also targeted by the vsiRNAs (Xu and Zhou, 2017). Hence, vsiRNAs that target genes involved in RNA silencing, viral immunity, and hormone signaling pathways are crucial determinants of the disease outcomes.

From the perspective of hosts, the silencing effects of vsiRNAs on its inherent defense mechanism would be counter-productive and such vsiRNAs would be selected against in the due course of evolution. On the other hand, from the pathogen’s perspective this could be a systematic counter-offensive strategy. This latter idea is further reinforced from the studies involving RSV infecting the vector plant hopper and rice (Yang et al., 2018). Although host-dependent, differential vsiRNA profiles are generated when RSV infects the planthopper or rice, careful scrutiny of the vsiRNA target genes in both the organisms reveals that most target genes are involved in defense response pathways. RSV-derived siRNA targeting the expression of trypsins and peritrophic matrix protein of plant hopper favors the replication of RSV whereas the target genes of rice such as that encode protein kinases, mannose binding lectin family proteins might facilitate the pathogenicity of RSV in rice (Yang et al., 2018). Lending further credence to this hypothesis, vsiRNAs of TYLCV effectively silence the host lncRNA (*SILNR1*) in the susceptible cultivar whereas manifestation of resistance trait of a tomato cultivar is due to the deletion of vsiRNA target site in *SILNR1* (Yang et al., 2019). Similarly, vd-siRNAs of *PSTVd* targets multiple callose synthase genes of the host (*CalS11-like* and *CalS12-like*) thereby affecting the callose deposition in pollen mother cells disrupting not only the reproductive pathways of hosts but also augmenting the entry point of secondary pathogens (Adkar-Purushothama et al., 2015).

## Perspectives and Concluding Remarks

Plant–virus interaction at the sRNA interface being a very intricate process, vsiRNA-mediated regulation of host transcriptome cannot be overlooked as an outcome of fortuitous pairing between sRNAs and host mRNAs. Comparably, in the world of fungi–plant interactions, gray mold fungi-(*Botrytis cinerea*)-derived sRNAs selectively target immunity-conferring genes of hosts, Arabidopsis and tomato (Weiberg et al., 2013). A unique strategy employed by CaMV divulges that accumulation of vsiRNAs from its genomic leader sequences enhanced the viral titer and hence the viral replication. Blevins et al. (2011) speculated that the vsiRNAs from leader sequences might serve as decoys, diverting the host RNA silencing machinery from targeting the viral promoter or coding regions. A parallel can be drawn with the findings from a human adenovirus wherein highly structured virus-associated RNA (VA-RNA) sequesters Dicer and counteracts interferon-mediated antiviral defense (Andersson et al., 2005). Hence, the possibilities of deployment of decoy as a strategy to counteract host defense mechanism in both animals and plants are common. However, no research evidences to prove the occurrence of such phenomenon and hence, it is a key area of future research.

Similarly, non-coding RNAs of viruses act in “*trans*” and function as molecular “sponges” sequestering the host protein factors so that host cellular signaling is modified in favor of virus persistence and or establishment (Miller et al., 2016). Beet necrotic yellow vein virus (BNYVV) encoded viral suppressor of RNA silencing, p14, is functionally complemented by the virus derived ncRNA (ncRNA3) which promotes the systemic movement of viral particles whenever p14 loses its function due to mutations (Flobinus et al., 2016). Thus, it is far from uncommon to observe sRNAs as effective regulators of host gene expression in pathogen–plant interactions. The effectiveness of vsiRNA in silencing a host transcript is determined by many factors, besides the perfect or permissible level of sequence complementarity to the target transcripts. Hence, the role of other non-coding RNAs of viral origin requires thorough investigation so that novel modes of action of viral sRNAs could be deciphered.

vsiRNAs originate from the hotspots of the viral genome hence, the entire genome is not equally accessible for host sRNA-mediated antiviral RNA silencing. However, it is unclear if the host RNA silencing machinery alone determines the family of siRNAs produced as antiviral agents. Alternatively, viral genomes would have acquired secondary structural features in due course of evolution to evade the host RNA silencing machinery. Evidences indicate little correlation between vsiRNAs of viral hot spot and antiviral resistance hence, it was suggested that those abundant vsiRNAs might participate in other biological functions (Szittya et al., 2010). Also, vsiRNAs from hot spots do not exhibit greater silencing efficiency compared to the vsiRNAs from non-hot spot regions, indicating only a fraction of abundant vsiRNAs are incorporated in RISC containing AGO (Xia et al., 2014). Alternatively additional pathways such as translational inhibition may contribute to the gene silencing besides mRNA cleavage (Wang et al., 2016). Therefore, the biological questions pertaining to the origin of vsiRNAs and their efficacy in conferring viral or host gene silencing warrants a thorough investigation.

Viral strand biasness in generating more sense-vsiRNAs over antisense-vsiRNAs could not be explained if they were generated by DCL from a dsRNA precursor. Recent research has shown that complementary vsiRNAs are sequestered by viral genomic RNAs during gel electrophoresis. Application of fully denaturing formaldehyde PAGE (FDF-PAGE) technique revealed that long dsRNAs are precursors of vsiRNAs (Harris et al., 2015). Moreover, factors like sequestration of vsiRNAs by viral suppressors of RNA silencing, degradation by sRNA nucleases *in vivo*, chemical and structural modifications *in vivo* are some of the important gray areas that require considerable attention of the researchers. It requires the application of tools that dissect protein: RNA interactions *in vivo* to uncover the functionalities of the vsiRNAs.

Features like 5′terminus nucleotide of siRNA also play important role in the efficacy of RNA silencing activity of vsiRNA. AGO 1 preferentially incorporates siRNAs with nucleotide “U” at 5′ terminus. Furthermore, recruitment of vsiRNAs onto AGO and RISC lacking proper ‘slicer’ activity also diminishes the cleavage potential of the sRNAs. Above all, the degree of vsiRNA activity against the host transcripts is also influenced by its relative abundance (Qi et al., 2009). Differential expression levels of siRNAs was observed in plants naturally infected with Y sat RNA and transgenic plants expressing inverted repeats of Y sat RNA. Thus, siRNA expression levels corresponded with the differential appearance of symptoms (Shimura et al., 2011). This observation was further demonstrated when constitutive expression of ToLCNDV-derived vsiRNA as short hairpin RNA caused off-target silencing of tomato transcripts. Whereas, a long hpRNA, which potentially forms many vsiRNAs-including the one expressed in shRNA, did not evince any off-target effects (Praveen et al., 2010).

Enormous quantum of data is being generated concerning the nature of vsiRNAs and vd-siRNAs encompassing wide variety of plant and pathogen species. It is also possible for vsiRNAs to act on host chromosome in TGS manner involving RNA directed DNA methylation. However, little research evidences are available to prove this probability, even though sequence-specific epigenetic modification of viral genomic regions mediated by siRNAs is common (Bond and Baulcombe, 2015; Lewsey et al., 2016). Development of plant exclusive vsiRNA database (PVsiRNAdb) with 282,549 unique sequences of vsiRNAs is a useful resource to explore the complex relationship between host and virus and improve deeper understanding of viral genomics (Gupta et al., 2018). However, understanding the mechanism of vsiRNA action on host mRNA is still a challenge for researchers. Also, the exhaustive collection of vsiRNAs could be used to reconstruct the virome and to investigate the interactions between virome and plant RNA silencing machinery (Pooggin, 2018). Large scale application of functional genomics tools such as target mimicry, short tandem target mimic, miRNA sponge to investigate sRNA inhibition will certainly facilitate in this endeavor (Teotia et al., 2016).

Functional miRNAs derived from viral genomes are generally identified in DNA viruses (Cullen, 2009) not in RNA viruses replicating in cytoplasm as the protein machinery necessary for miRNA generation are located within nuclei. However, RNA viruses replicating in cytoplasm have also been known to encode functional miRNAs (Rouha et al., 2010; Sankaranarayanan et al., 2020). Biological significance of virus-derived miRNAs in animal system is known, however, the relative advantage of expression of plant virus-derived miRNAs is yet to be unraveled. Vast majority of the plant infecting viruses are RNA viruses and therefore harboring nucleotide sequences that are prone to the host-derived DICER or RNase would be disadvantageous. In this context, expression of miRNAs by some plant RNA viruses poses many questions. Is it a counter-offensive strategy to tackle the plant’s antiviral defense system? Or is it a regulatory mechanism to modulate the viral gene expression? However, it is speculated that plant virus-derived miRNAs are effective counter-defense strategy by way of regulating host transcripts to the advantage of pathogen. Viral-derived miRNAs are implicated in regulation of viral gene expression as in HCRSV’s instance thus the molecular intricacies of sRNA-mediated viral gene regulation requires to be thoroughly investigated. It is suffice to state that viruses effectively employ two-pronged strategy involving viral suppressors of RNA silencing and small RNAs as effective modulators of host defense mechanism to increase its survival.

## Author Contributions

SVR, SY, PG, SC, SW, and HRP performed the data mining and drafted the various sections of the manuscript. All the authors have read and approved the final version of the manuscript.

## Conflict of Interest

The authors declare that the research was conducted in the absence of any commercial or financial relationships that could be construed as a potential conflict of interest.
